# Recent Works on Pathological Anatomy

**Published:** 1834-04-01

**Authors:** 


### Medico-Chirurgical Review,

No. XL.

JANUARY 1, 1834, TO APRIL 1, 1834.

### I.

### RECENT WORKS ON PATHOLOGICAL ANATOMY.

I. Illustrations of the Elementary Forms of Diseases. By *Robert Carswell*, M.D. Professor of Pathological Anatomy in the University of London, &c. Fasciculus IVth. Melanoma. Longman's, London. 1834.

II. Principles and Illustrations of Morbid Anatomy, &c. By *J. Hope*, M.D. F.R.S. &c. Parts IX. and X. for January and March, 1834. Whittaker, and Co. London, 1834.

III. Anatomie Pathologique du Corps Humain, &c. Par *J. Cruveilhier*, Professeur d'Anatomie à la Faculté de Médecine de Paris, &c. Dix-huitiéme Livraison. A Paris, chez J. B. Bailliere, Libraire, 1833.

IV. The Second Fasciculus of Anatomical Drawings, selected from the Collection of Morbid Anatomy in the Army Medical Museum at Chatham. Folio, Nine Uncoloured Lithographic Plates, London, 1834.

The contemplation of four such works as these on pathological anatomy, published at one time and almost in the same country, is calculated to give rise to a train of reflections in the mind of the most dull and unthinking observer of the world of medicine. It is but a few years since this department was a desert; it is now the fairest province in the land of science. Could the venerable fathers of our art rise for one short hour from their graves, and observe the dissector and the draughtsman busy at their avocations—see their ancient systems and their toilsome observations overthrown by labours, which their infancy in philosophy and their natural prejudices consigned to the earth-worm and the mole—view the morbid anatomist deriding their dogmas and pitying their credulity—those primitive sages would hurry to the land of sprites, and quit without reluctance a new world, too wise or too insane to have aught of community with them.

Could they who drew the bow and fixed the arrow see where it has sped, they would probably own that they had been but blind and short sighted instruments, in the hands of that Power which guides the destiny and determines the progress of the human race. Bichat, Baillie, and the Hunters commenced the investigation of the anatomy of disease. Their

æra is scarcely passed away, yet the study on which they entered has already revolutionized the practice and the theory of medicine.

There are many who regard this with horror and dismay—more who look on with misgiving and suspicion. The former class of thinkers sigh at the downfal of Hippocrates and Galen. They cling to the doctrine of critical days, and hug the belief in critical things.* Fever is an entity, regulated, like the tides, by some mystic influence—rheumatism lasts for about six weeks—sarcocele, white-swelling, gravel, are good old terms with a good old meaning. These gentlemen, like the Tories of forty-five, have a hopeless attachment to the ancient dynasty—they drink to the king over the water, and damn the House of Hanover. They must have their way, and as Time, the great innovator, takes them to his bosom, their stools will be filled by younger and more ardent spirits.

We cannot say that we participate in the apprehensions of those who regard with suspicion the taste for morbid anatomy. It is a good fault to lean to the side of exactness and induction, a fault which rather breeds a sternness than a laxity of reasoning. Some amount of enthusiasm must be expected and excused in the application of a power which has worked such miracles already. Extravagant anticipations will in time be sobered down, and reason and sense are tolerably sure of prevailing in the long run. Pathological research has done and will do sufficient for our science, to compensate a thousand times the injury it ever can occasion.

Such are our own sentiments, and actuated by them we have endeavoured and shall still endeavour, to lay before our readers, as occasions offer, an abstract of what is done by labourers in this department. The very persons who object to the zealous cultivation of morbid anatomy cannot fail to perceive that they *must* keep pace with it. The race is going on, and the man who is unhorsed or who quits the saddle will never see the goal.

Leaving this train of general remark, we will now advert to the works before us. The subjects they embrace are melanoma—hypertrophy of the tissues of the intestinal canal—scirrhus and cancer—diseases of the uterus and the ovaria—of the kidneys, renal capsules, and the cerebellum—of the larynx and lungs, the small and large intestines, the heart and the great vessels. It must be owned that the variety and abundance of the viands require and perplex selection. It is so difficult and so tedious to amalgamate the opinions of different individuals, that we commonly prefer adverting to them in succession. This is the method we shall now adopt.

And first of Dr. Carswell. The subject of his fourth Fasciculus is MELANOMA. He observes that various and very different morbid products have been confounded with melanosis, as first described by Laennec. He thinks that those black formations or products which depend on a change in the process of secretion, and constitute an idiopathic disease, should be viewed as true melanosis; whilst those which originate in the accumulation of a carbonaceous substance introduced into the body from without, the action

* A highly respectable physician lately took us to task, in the Medical Gazette, for smiling at the doctrine of Critical Days. He will pardon us for declining to commence a controversy. The relatives of Dives would not have been convinced, even if Lazarus had left Abraham's bosom.

of chemical agents on the blood, or the stagnation of this fluid, may be grouped under the denomination of *spurious melanosis.* True melanosis is therefore considered as of one kind: spurious melanosis as of three: 1st. from the introduction of carbonaceous matter ; 2nd, from the action of chemical agents on the blood ; 3d. from the stagnation of the blood.

1. True Melanosis. This is a morbid product of secretion, of a deep brown or black colour of various degrees of intensity, unorganized, influenced in its form and consistence by external agents.

Its principal seat is in the serous and the cellular tissues. It is observed in the substance or molecular structure of organs. It is found in the blood contained chiefly in the venous capillaries, under circumstances which shew that it must have been formed in these vessels. The much greater frequency of melanosis in the grey and white than in the bay, brown, or black horse, is a circumstance which may be noticed here as favourable to the theory which ascribes the origin of this disease to the accumulation in the blood of the carbon which is naturally employed to colour different parts of the body, as the hair, rete mucosum, choroid, and other parts.

Dr. Carswell considers in order the physical, chemical, and anatomical characters of melanosis. The physical comprise the form, the bulk, colour, and consistence.

*Form.* This displays four varieties :—punctiform—tuberiform—stratiform—liquiform.

*Punctiform Melanosis.* "This form of the disease appears in minute points or dots, grouped together in a small space, or scattered irregularly over a considerable extent of surface. These appearances are most frequently seen in the liver affected with melanosis, the cut surface of this organ appearing as if it had been dusted over with soot or charcoal powder. When examined by the aid of a lens the black points sometimes present a stellated or penicillated arrangement, which, in some cases, can be distinctly seen to originate in the ramiform expansion of a minute vein filled with the melanotic matter. At other times this matter appears to be deposited in the molecular structure of this organ in a manner similar to that of the organizable part of the blood. In such cases it consist of the most minute points disseminated throughout the acini of the liver, which then assume a uniform grey aspect of various depths of shade, terminating in black." 2.

*Tuberiform Melanosis* is the most common. It occurs in most of the organs, and sometimes upon serous surfaces. In the former situation the tumors are generally globular; in the latter not unfrequently pyriform. They are usually found single in organs, and aggregated in cellular and adipose tissues ; perhaps they have never been found limited to one organ. The tumors are most numerous in the cellular and adipose tissues, and produce by their aggregation masses of great bulk. The tuberiform melanosis is always combined with the punctiform in the liver, lungs, and kidneys, while on serous membranes they are accompanied by the liquiform variety of the disease. The tumor may or may not be encysted. It is, perhaps, never found so in compound tissues or organs, as the brain, lungs, liver, and kidneys ; it is always so in the cellular and adipose tissues; and sometimes so on the surface of serous membranes.

*Stratiform Melanosis.* This occurs only on free surfaces, and presents two stages or degrees. In the first, the serous membrane on which it is deposited presents an appearance of having been painted or stained with a deep brown or black colour. In the second, the black deposit is more abundant, and forms a distinct layer on the surface of the serous membrane above which it slightly projects. The consistence of the matter thus deposited resembles in general that of jelly, and is inclosed either in a soft spongy cellular tissue, or fine transparent serous membrane of new formation; so that, when pressed, it feels pulpy, but is not removed by the finger or a scalpel passed over it, unless some force is employed. It is rare and limited in extent in man, but it is sometimes considerable in the horse, and is chiefly found on the peritoneum, pleura, and pericardium.

*Liquiform Melanosis.* It has in general been confined to natural or accidental serous cavities. Among the former, the cavities of the pleura and peritoneum furnish almost the only examples in which the liquid melanotic matter has been observed, and that too in very small quantity. The accidental serous cavities in which it has been found are those which constitute cysts, particularly in the ovaries.

*Bulk.* The quantity of melanotic matter is often considerable. The largest masses are found in loose cellular tissue, and are always composed of a number of smaller ones; but single masses of the largest size have been discovered in the liver. Masses have been found in the former situation in the horse, weighing from twenty to forty pounds.

*Colour.* This ranges from black to brown. The deep black colour and glossy aspect are more frequently met with in inferior animals than in man, and in both these appearances are most marked when the deposit exists in the form of a firm tumor.

*Consistence.* It is entirely dependent on the nature of the part in which the black deposit is retained. It is never found solid in serous cavities—in the tumors attached to the serous covering of those cavities, it is liquid or gelatinous—it is occasionally fluid in loose cellular tissue—in the cutis it is nearly as hard as cartilage—it is of medium consistence in the lymphatic glands and in the brain, &c. At an indefinite period of its formation, the solid melanotic tumor is observed to lose its consistence, and ultimately to become converted into a soft or almost fluid mass. This softening process is analogous to that which occurs in many morbid growths, especially in those of the fungoid character. Dr. Carswell believes that it is effected by the destruction of the tissues which surround or are inclosed within the melanotic tumor, and the simultaneous effusion of serosity. Inflammation rarely accompanies this process, and when ulceration and sloughing occur, they appear to be chiefly owing to the melanotic matter compressing or obliterating the bloodvessels of the tissue in which it is contained.

"The following may also be enumerated as physical characters of the melanotic matter. This matter is quite opaque, and has no marked odour or taste. In its natural state, or when mixed with water, exposed to the air it becomes dry, brittle, and pulverizable, and does not emit the odour of putrefaction until after a long period. When burnt it swells, gives out a great deal of smoke, a marked empyreumatic odour, and is converted into a carbonaceous substance." 5.

*Chemical Characters.* According to Barruel, melanosis of the human subject is essentially composed of the colouring matter of the blood, united with fibrine, both of them " se trouvant dans un état particulier,"—three distinct kinds of fatty matter—and a considerable quantity of phosphate of lime and iron. From the results of the various analyses it is obvious that the melanotic matter is essentially composed of the constituent elements of the blood, and probably the substance which confers its colour is a highly carbonized principle, having much analogy to the colouring matter of the blood.

*Anatomical Characters.* When a single encysted tumor is divided, and a quantity of the melanotic matter removed by pressure and ablution, it is found to be traversed in every direction by a multitude of fine filaments and lamellæ connected with the capsule at the periphery.

"When a number of melanotic tumours are grouped together, they are included in a common capsule, and separated from one another by their respective coverings, and portions of cellular tissue contained in the angular spaces sometimes left between them. It is in these filamentous and cellular tissues alone that bloodvessels or nerves are to be seen. Minute arteries and veins may be observed ramifying in both, but they never pass beyond the limits of these tissues. Large branches, and even trunks of arteries and veins, are sometimes found passing over the surface, or included in the aggregated masses of melanotic tumours." 5.

The cellular tissue is usually not abundant in quantity. In some rare instances it exceeds the melanotic, but this is when the latter is deposited in fibrous, carcinomatous, or erectile structures, constituting compound tumors.

" Melanosis is more frequently combined with carcinoma than with any other disease, but there is no similarity of nature in these two diseases, their anatomical, physical, and chemical characters being totally different; several varieties of the former are highly organized, while the latter is an unorganized substance, injurious only from its quantity, the number of organs which it affects, its situation and mechanical operation." 6.

We do not feel so confident as Dr. Carswell seems to be upon this point. The frequent combination of melanosis with carcinoma is suspicious—the invasion of many organs in succession by the former is suspicious also—the tendency of melanotic tumors to softening and to destruction by ulceration and by sloughing, is another feature calculated to encourage the idea that too near a relationship exists between melanosis and carcinoma. We would pause in the present state of our knowledge on these subjects, before we would subscribe to the positive assertion that melanosis is only injurious from quantity and situation. We require more facts, more time, more observers, to determine a circumstance of this importance.

Spurious Melanosis. This, it will be recollected, was divided by our author into four varieties. The first is, *From the Introduction of Carbonaceous Matter.* It occurs only in the lungs.

*Physical Characters.* Both lungs present one uniform black carbonaceous colour, in which the bronchial glands participate. The pulmonary tissue is more or less indurated and friable, infiltrated with black serosity, and broken down in several parts into irregular excavations, sometimes of considerable size.

*Chemical Characters.* A minute analysis was made by Dr. Christison, of a quantity of the black matter taken from a patient who died of this disease in the Royal Infirmary of Edinburgh. Waiving the particulars, we may content ourselves with stating, that a portion of black powder left after the action of concentrated nitric acid boiled on the black matter, gave the ordinary products of the distillation of coal. A gas of the same quality was procured, and likewise a naphthous fluid, holding in solution a crystalline principle, analogous to, if not identical with naphthaline.

Dr. Carswell considers it as clearly proved by this interesting case and satisfactory analysis, that certain forms of black discolouration of the pulmonary tissue originate in the inhalation of the carbonaceous product of ordinary combustion.

The second form of spurious melanosis is that from the *Action of Chemical Agents on the Blood.*

"The black discolouration of the blood which belongs to this division of spurious melanosis, is produced by the operation of an *acid* chemical agent. It is, consequently met with only in those organs in which this agent exists as a healthy or morbid product, or to which its influence occasionally extends. Hence, as the stomach is the only organ in which an acid fluid is formed, and is, at the same time, a healthy product, this kind of black discolouration of the blood is no where so frequently observed, so conspicuous, and so extensive as in this organ.

The same discolouration of the blood occurs also in the intestines from the anormal formation of a fluid and gaseous acid product. It is owing to the proximity of the peritoneum to these normal and anormal acid products, that blood situated beneath this membrane, on its free surface, or in its cavity, undergoes so often the change of colour in question; and it is owing to the same circumstances of situation, that portions of the liver and spleen are so frequently found to present the same black colour.

The black discolouration of the blood may be effected during life or after death; but before it can take place during life, the blood must have ceased to circulate. This fluid may be contained in its proper vessels, or effused into the cavity of the stomach, intestines, or peritoneum." 7.

*Colour.* This varies from a dull yellow to that of bistre, or soot. The brown and black tints are most frequent in their occurrence—the yellow and orange are seldom seen except in the mucous membrane of the stomach.

*Forms.* They are determined by those of the organs in which the blood is contained; they are the punctiform, ramiform, stratiform, and liquiform.

The punctiform and ramiform black discolourations of the blood have their seat in the capillaries and the veins, and are best seen on the internal surface of the stomach, where they most frequently occur. They always occupy that part of the organ in which the gastric acid is contained after death. They present an appearance similar to what would be produced by injecting the capillaries and veins of a portion of the stomach with a mixture of chocolate or soot and water. Their extent is proportioned to the quantity of gastric acid—the degree of discolouration to the amount of blood contained in the vessels. In the stomach this is always accompanied with the chemical dissolution of its coats.

The ramiform black discolouration of blood from the presence of an acid is seldom met with in the intestines, but the punctiform is not uncommon. The latter takes place in the capillaries of the villosities, and in those around the orifices and basis of the follicles. When the villosities are the seat of the discolouration, the surface of the mucous membrane appears as if it had been dusted all over with fine powdered charcoal, which gives to it a deep grey or slate colour. The discolouration may occupy the orifice of a number of contiguous isolated follicles, or of the aggregated follicles, and give rise to an appearance resembling acne punctata; or it may surround the orifice or basis of the isolated follicles, when it has an annular form.

The ramiform discolouration is met with occasionally on the peritoneum, in cases of chronic tubercular peritonitis. The tubercles are surrounded by a dark ring, or a multitude of minute vessels filled with black blood, either grouped close together, or having a stellated arrangement. The tubercles, if small, are thereby greatly obscured, and the peritoneum appears as if spotted with a deep brown or black pigment.

The *stratiform* black discolouration is most frequently observed on the peritoneum, in its subcellular tissue, or in false membranes when blood has been effused upon the former, or into the latter. The discolouration varies in extent—and in its tint, ranging from black to brown or even red.

The *liquiform* black discolouration of the blood is produced by the effusion of this fluid into the cavity of the stomach, intestines, and peritoneum. It forms the black vomit and melœna. In the large intestines and in the cavity of the peritoneum its blackness depends, perhaps, more frequently on the presence of sulphuretted hydrogen than on an acid.

*Black Discolouration from Stagnation of the Blood.* This has long been known to follow retarded or interrupted circulation. There are, according to Dr. C., only two organs, the respiratory and digestive, in which this change of the blood bears any resemblance to true melanosis. In the lungs the black discolouration of the blood accompanies all those diseases which impede mechanically the capillary circulation, and render the function of respiration imperfect. It may pervade the whole lungs, a limited portion of their tissue, or a multitude of spots or minute points. It is never so intense when general as when partial. It occurs not only much more frequently, but has a deeper tint in the summits than in any other portion of the lungs.

The blood in this state of discolouration constitutes the "Matière noire pulmonaire" of Laennec. It may exist either alone, or in combination with accidental products, and presents the three following forms, viz. the punctiform, ramiform, and maculiform. Of the two former we need say nothing. The latter is seldom met with except in the upper lobe of the lung in old persons.

"A portion,—most frequently the summit, or the whole of this lobe,—is often found in them studded with dark grey, purple, blue, and black spots. The pulmonary tissue contains little or no air, is firm and œdematous, or quite hard and somewhat dry. Masses of a putty-looking or cretaceous substance, of fibrous, cartilaginous, or osseous tissues, are generally seen lodged in the darker portions of it, and the bronchi and bloodvessels are either much compressed or obliterated. This kind of discolouration follows the cure of tubercular phthisis, and is obviously the consequence of the interrupted state of the venous circulation in the affected part of the lung." 10.

We fear we know too little of the actual condition of the lung *after* the cure of tubercular phthisis to permit us to decide very confidently on the affection of the tissues left behind. Dr. C. remarks that the grey slate colour of the indurated pulmonary tissue which forms the boundaries of tubercular excavations is of the same nature as the preceding.

We need not touch on black discolouration of the bronchial glands. It is sufficiently familiar.

Black discolouration of the blood from stagnation in the digestive organs, is confined to the villous and follicular structures of the mucous membrane.

We cannot close the notice of the letter-press of this fasciculus, without expressing our high opinion of the philosophic and exact description of melanosis which the author has presented. We think he has divested himself of that leaning towards theory which he displayed in the earlier portions of the work—a leaning which we took the liberty to criticize, as peculiarly inappropriate to so rigorous a study as morbid anatomy.

The plates are four in number and contain 23 coloured figures. We do not think them equal to some of the same author's, in other parts of the same work, but they are not deficient in pictorial beauty. It would be useless to compose a catalogue of drawings not presented to the inspection of our readers, but a hasty glance at the subjects represented may not be totally devoid of interest.

The first plate displays true melanosis of the liver, the lung, the skin, and the cellular tissue, the two latter instances being taken from the horse; it also shews a compound melanotic tumor. This consists chiefly of fibrous tissue, between the lamellæ of which the melanotic matter is deposited.

The second plate contains seven figures illustrative of a remarkable case of melanosis that occurred in an old man, who was taken to the Hôtel Dieu. Brown or black coloured tumors were contained in the brain, the epiploon, and ileum. The tumors were of the compound character, being composed of true melanosis, combined with carcinoma and erectile tissue.

In the third plate are delineated a portion of the liver affected with melanosis and carcinoma, the latter being much more abundant than the former —the black discolouration of the lungs from the inhalation of carbonaceous matter—the black discolouration of the same organ from stagnation of the blood—the same in the lung and bronchial glands from a mechanical obstacle to the return of the venous blood—and, finally, the black discolouration of the blood in the villous and follicular structures of the intestines.

The fourth plate exhibits several forms of black discolouration of the blood from the chemical action of the healthy gastric acid, fluid, and gaseous acid products formed accidentally in the intestines.

We cannot quit the fasciculus before us without expressing the gratification we have derived from its perusal.

---

The ninth fasciculus of the work of Dr. Hope is devoted to Hypertrophy of the Tissues of the Intestinal Canal, to Scirrhus, and to Cancer. In the tenth, Diseases of the Ovaria are considered, and Diseases of the Uterus commenced.

*Hypertrophy of the Tissues.* Dr. Hope describes, in succession, hypertrophy of the submucous, the muscular, and the subperitoneal tissues. The principal seat of this affection is certainly the submucous; it is found in a large proportion of cases of chronic diarrhœa, in which the mucous membrane has become diseased, and especially in the vicinity of old ulcerations. There is no part of the canal below the diaphragm in which hypertrophy of the submucous cellular tissue has not been observed; but it occurs principally in the parts most liable to chronic inflammation of the mucous membrane, namely, the stomach, the rectum, the colon, and the end of the ileum. Hypertrophy of the muscular coat is found principally where that coat is naturally of the greatest thickness, namely, in the pylorus and pyloric third of the stomach, in the cardia, the rectum, and the colon.

"Contraction of the pylorus is occasionally attended with enormous dilatation of the stomach, which has been known to reach even to the ossa pubis *(Andral, Clin. Med.)* Under these circumstances, the walls are sometimes attenuated, and sometimes of natural thickness. The dilatation arises from distension of the organ by accumulations of food which cannot pass through the pylorus. After the lapse of several days, the stomach, distended to the extreme, relieves itself by disgorging its contents; and hence, says Andral, arise those vomitings, so remarkable for their extreme copiousness, which supervene from time to time (every eight or ten days, for instance) in individuals labouring under contraction of the pylorus. When the contraction proceeds from carcinomatous tumours, the vomiting ceases when the ulceration of the tumour leaves the orifice free; but the symptom recurs in proportion as the tumour is regenerated. It occasionally happens that similar dilatation of the stomach takes place when the pyloric orifice is enlarged; an anomaly which is explained by the thickened walls of the viscus having lost their tonic contractile power, whence they allow accumulations to take place, as in an inert bag. When the pylorus is much thickened, a tumour may sometimes be felt externally; and when the whole stomach is diffusely thickened and contracted, without forming any particular tumour, an unusual resistance is, in some cases, perceptible over the whole epigastrium.

In the small intestines, hypertrophy of the submucous tissues is rare, and it is generally confined to a particular spot, of small extent. Sometimes the symptoms of stricture are produced by it, and increase from time to time to those of strangulation, of which the patient, after several relapses, usually dies. The intermittent nature of the symptoms of strangulation is ascribed by Andral to temporary tumefaction of the mucous membrane of the contracted portion; but it is also certain that the mere circumstance of distension of the gut above the stricture will, in contractions of a certain form, tend to close the passage." 215.

In the colon, hypertrophy is more common at its two ends than in the middle, because, at those ends, the mucous membrane and follicles are more subject to disease. The thickening is in general attended with contraction of the gut; but sometimes the reverse takes place, and the walls, having lost their contractility, become dilated into a cyst with thick and hard parietes, forming adhesions with the contiguous parts, and presenting externally the feel of an abdominal tumour.

Stricture of the rectum or of the colon is in general nothing more than hypertrophy of the submucous cellular tissue, succeeding to irritation and thickening of the mucous membrane itself. We have seen an instance of contraction of the rectum, resulting from the cicatrization of ulceration of the mucous membrane.

Condylomata around the anus are formed by hypertrophy of the submucous cellular tissue, covered with the thickened and more or less injected mucous membrane, within the gut, and by the cuticle external to it. There are many instances of condylomata, in which the elevations are circular and flat upon their surface, and which essentially depend on interstitial deposition into the texture of the cutis itself. Of this we have satisfied ourselves in many venereal cases. When such condylomata are superficially ulcerated, as they often are, they constitute one variety of the elevated ulcer, or the condylomatous sore, and are followed by secondary symptoms.

Hypertrophy of the intestinal canal occurs most frequently between the ages of thirty-five and sixty-five, and is very rare between puberty and thirty-five. It is not uncommon in children between the ages of four and twelve, when of unhealthy constitutions and subject to chronic diarrhœa.

Dr. Hope proceeds to the consideration of scirrhus. The distinction between hypertrophy and scirrhus, though generally free from difficulty, is sometimes difficult. Occasionally they merge into each other.

"Hypertrophy is apt to pass into scirrhus, because the local irritation, while it gives rise to an increase of the natural nutritive function, also favours the separation of cancerous matter from the blood, if a tendency to the disease pre-existed in the system. But the morbid deposition may likewise take place as a primitive lesion, wholly independent of previous hypertrophy; and the resulting appearances may be so similar, for a time to those of hypertrophy, that the discrimination can only be effected by collateral evidence. Namely, in cancer, the disease is commonly of limited extent; in hypertrophy many feet of the canal are often affected; cancer is more confined to the stomach and rectum than hypertrophy, it is attended with greater thickening of the walls, the muscular coat is more frequently implicated, and there is in many instances a coexistence of cancer in other organs, particularly the liver. When the morbid deposition has advanced so far as to become divided into lobules by fibrous intersections, or hollowed into cells or areolæ filled with gelatinous matter, these physical characters alone are sufficient to mark the cancerous nature of the disease." 217.

There is a circumstance which should always be remembered in pursuing investigations of this nature. Morbid growths ulcerate and are incurable without being actually scirrhus. They form the link between the latter and simple ulcerations. Thus the eyelids and the sides of the nose of old people are subject to a disease, which begins as a tubercle in the skin or the subcutaneous texture.

Dr. Hope passes over the slighter varieties of scirrhus, to expatiate on that form which has been denominated by Cruveilhier *areolar gelatiniform cancer.* It consists of a transformation of the affected tissue into a fibrous alveolar web, filled by a sort of transparent jelly. In the highest degree of the alteration, all kind of organization has disappeared: no trace of vessels is longer to be discovered, and all the tissues, however heterogeneous, seem brought to one uniform morbid type. Cruveilhier believes that in this, as in all other instances of organic degeneration, the primitive seat of the deposition is the cellular tissue.

It occurs in two forms; 1, in disseminated masses, varying from the size of a millet-seed to that of a turkey's egg, or more; 2, uniformly infiltrated throughout the substance of the organ affected.

1. In the disseminated form, the disease sometimes presents a few detached prominent tumours, with heads broader than their bases; they may attain the size of turkey-eggs. Sometimes it exhibits an infinite number of small, contiguous, and coalescing tumours, diffused over a greater or less extent of surface. Whatever be their size and number, whether isolated or coalescing, they are soft, spongy, semitransparent, and, when examined attentively, are seen to consist of areolæ infiltrated with gelatiniform matter. They are considered by Cruveilhier to be nothing more than mucous papillæ, prodigiously developed by the morbid deposition.

2. In the infiltrated form, the gelatinous matter is deposited uniformly throughout the substance of the organ, the part then maintaining its general shape, but undergoing a remarkable thickening. In the stomach, the submucous tissue is usually affected before the mucous. Sometimes the deposite commences separately in the muscular and submucous textures; more commonly it is propagated from one to the other by continuity of tissue, the muscular coat, according to Cruveilhier, becoming hypertrophous, and divided into fasciculi by fibro-cellular partitions, along which the gelatinous deposition spreads.

"Areolar gelatiniform cancer, though found in the rectum, the cæcum, and occasionally in the ileum, is in no part of the alimentary canal so frequent as in the stomach. In this organ it exhibits, in common with the other varieties of cancer, a predilection for the pylorus; but it extends its ravages on each side, generally to a limited degree on the duodenal side, but often over a lage space on the side of the stomach of which it may pervade a third, a half, and even the whole. After the pylorus, the lesser curvature is the part most liable to the first attack of the disease. The exterior of the organ often presents a lumpy surface, from the unequal contraction of the muscular coat, and the absorbents are occasionally seen ramifying on it, charged with cancerous matter, and presenting the appearance of knotty cords of a whitish or translucent greyish colour. The veins also are sometimes charged with the same matter." 221.

We must introduce another quotation, and that a rather long one.

"The present species of cancer is an extremely obscure disease, in reference both to its local and its constitutional symptoms. The only characteristic local signs which it offers are those of a mechanical obstacle to the passage of the food. In the absence of these, and of an external tumour or induration, the symptoms are such as cannot be distinguished from those of chronic inflammation or irritation; and patients are found to live and carry on digestion for a considerable period, when half, three-fourths, and even the whole of the mucous membrane is deficient! The constitutional symptoms, according to Cruveilhier, acquire the characters of cancerous cachexy more slowly than in any other variety of cancer; they exhibit less of general re-action and irritation, and the deposition itself seldom takes place, simultaneously or successively, in any considerable number of different parts.

The reason why areolar gelatiniform cancer is of so sluggish and chronic a nature appears to me to be, that it possesses exceedingly little vascularity; whence it has no intrinsic power of extension and propagation—in this respect forming a strong contrast with encephaloid cancer, which, by its high vascularity, effects its own reproduction with surprising celerity.

It is rare, according to my observation, to see well-marked encephaloid cancer affecting the submucous tissues of the alimentary canal, the ordinary form of the disease in these parts being scirrhus, under which term I include *areolar gelatiniform cancer*. Sometimes, however, we see an opaque whitish matter deposited in spots in the midst of hypertrophy of the submucous tissue or of the muscular coat. Thus, it is seen in connexion with an encephaloid tumour, adherent to the peritoneum.

The observations which I have made on vomiting, in consequence of contraction of the pylorus by hypertrophy, apply equally when the obstacle is occasioned by cancer. But a mechanical obstacle is not the only cause of vomiting in cancer; for, independent of any obstruction, it may result from the morbid irritability of the stomach in the advanced stage of the disease, and especially when the surface of the organ is denuded by ulceration. In this case, the vomiting, instead of being periodical and affording relief, comes on promptly after the introduction of ingesta, and the straining is attended with pain, sometimes of great severity. At this period of the disease, the matter ejected is generally spotted, streaked, or freely intermixed with a substance resembling coffee or chocolate-grounds, or lees of wine. This is blood extravasated from the disorganized surface, and changed by exposure for a sufficient period to the action of the gastric juice. It generally augurs a near approach of the fatal termination. I have already shown that a similar matter may be a product of exhalation from the mucous membrane in cases of chronic gastritis, and that it even takes place in the vessels themselves when the mucous membrane is removed by softening. The same dark matter may appear in the excretions when cancer has affected the intestines, and, by ulceration, led to hæmorrhage. In this case, the discolouration of the blood is effected by the intestinal fluids and gases, which, like the gastric juice, have acid qualities." 223.

We feel inclined to believe that, in cases of cancer of the stomach, diarrhœa and black dejections occasionally supervene, independently of affections of the intestines themselves. We lately saw a case of ulcerated scirrhus of the cardiac end of the œsophagus and contiguous portion of the stomach, in which there had been diarrhœa, with melæna, previous to the patient's death. There was no disease of the mucous membrane of the bowels, but a quantity of grumous liquid was contained within the stomach. Probably the blood and the discharge from an ulcerated scirrhus of the cardia, acted on by the acid of the stomach, would prove of itself a sufficient cause of irritation to the bowels to occasion diarrhœa. Such would appear the explanation of the circumstances, in the case to which we have alluded.

Dr. Hope next proceeds to diseases of the peritoneum. His glance at them is so hurried, that we need not follow him. From these diseases he passes to the consideration of external cancer. The remarks on softening of the scirrhous tumour are deserving of attention.

"At a certain period of its progress the scirrhous tumour softens in its interior. The softening is not the result of inflammation, but of an obstructed state of the circulation, resulting sometimes from congestion, and sometimes from partial compression of the vessels by the unequal development of certain portions of the tumour, whereby the circulation through other portions is intercepted. The function of nutrition may thus be suddenly modified or wholly suspended over a large extent; and hence we sometimes see firm tumours undergo complete softening with surprising rapidity. The softened portion consists of sloughy matter, held loosely together by remains of cellular tissue and blood-vessels, and intermixed with a muddy serous fluid of variable consistence, and occasionally with blood. Hæmorrhage, however, is much less frequent in scirrhous, than in encephaloid tumours.

From the explanation now given, it will be apparent that softening does not necessarily commence in the centre of the tumour, but at any part, the circulation to which happens to be intercepted. The centre, in fact, occasionally presents a nucleus of such density as to be the last to undergo softening; for, not only is the vascularity of scirrhus less in proportion to its degree of induration, whence there is an inferior *direct* tendency to softening; but the circumstance of the density renders the circulation less liable to be intercepted by unequal compression." 227.

Dr. Hope remarks that the circumstances of their development exercise a very important influence on the character of scirrhous tumors. The presence or absence of compression, and the nature of the tissue in which they are seated will probably, when properly considered, resolve into a few simple principles many of the apparent inconsistencies and anomalies which perplex this subject. Dr. H. adverts to the difficulty of distinguishing with any precision the malignant from the non-malignant growths. He quotes the opinions of Mr. Lawrence and M. Andral, opinions which display, without diminishing the difficulty. Like a philosophical observer, he has made it his object to offer accurate delineations and descriptions, rather than to venture, without a clear perception of his path, into the misty regions of generalization.

### Diseases of the Ovaria.

Dr. Hope makes some remarks on the physiology of the ovaria, and on conception. Leaving these, we may pause for an instant to glance at chronic inflammation of the ovaries.

This is generally attended with little or no pain, and is therefore insidious. It occasionally converts the ovarium into a sac full of pus, the quantity amounting in one instance to twenty pints. The sac sometimes becomes softened and perforated, and the pus is either discharged into the sac of the peritoneum, exciting fatal peritonitis; or, what is more common, into the uterus, vagina, bladder, or intestines. In such cases recovery is not uncommon. Dr. H. saw a case in St. Bartholomew's, under Dr. Latham, where two pints were passed suddenly *per vaginam:* the tumor subsided at the same moment, and the patient recovered.

The description of ovarian cysts is concise but good. Our author passes in review the simple cyst—the multilocular cyst—their various contents—and, finally, malignant disease of the ovaria. He conceives that our means of diagnosis are not sufficiently distinctive, whilst the diagnosis itself is a matter of importance. In the early stage, the diagnosis from ascites is, for obvious reasons, not attended with much difficulty. We recollect a curious and rather ridiculous instance of mistake. A young lady had a tumor of about the size of a large fist, in the right iliac region. It was moveable, but not beyond the medial line—firm—apparently issuing from the pelvis. There was numbness in the right lower extremity, and a tendency to œdema in that foot. The health was indifferent—the catamenia were said to have been absent for a month or two. A hospital surgeon who saw the case pronounced that it was ovarian tumor—a physician coincided with him. The tumor increased in size, and began to occupy the centre of the abdomen. The surgeon was examining it one day when he felt a motion of no dubious character. The young lady was pregnant. After delivery no tumor could be found. The following hints on diagnosis are useful.

"When the tumor fills the whole abdomen, the diagnosis is more difficult: here, however, percussion affords excellent, and, with ordinary care, conclusive signs. In ascites, the intestines float upon the fluid, and the resonance on percussion is hollow in the most elevated situations—and invariably so in the umbilical and epigastric regions. (Piorry's ivory Plessimeter is here preferable to the hand.) On the contrary, the ovarian tumour being developed *in front* of the intestines, which it forces back, the most prominent part of the tumour is always dull on percussion. Further, to a practised ear, the dulness on percussion of an encysted dropsy is much greater than that of ascites; since, in ascites, the layer of fluid before the intestines is never so thick as to prevent a certain degree of resonance from being elicited by firm and smart percussion. Again, fluctuation is more distinct in ascites than in ovarian dropsy, unless much tympanitic tension coexist with the ascites—a very common case: but here, fortunately, the high degree of resonance affords an unequivocal diagnostic sign. In ovarian dropsy, the neck of the uterus is usually drawn up out of reach. The general symptoms also are different, ascites being almost always connected with some old organic disease of the liver, heart, or kidneys, and attended with infiltration in other parts; while ovarian dropsy may exist independent of them, and may even be compatible with a perfect state of the general health.

The single gelatinous cyst cannot be physically distinguished from the single serous cyst—the feel and fluctuation of both being the same; but the multilocular cyst may often be distinguished from the unilocular, by its inferior degree of fluctuation, and, still more, by its uneven surface perceptible through the abdominal parietes or through the vagina and rectum. Encephaloid ovarian tumours may often be detected by their uneven, or unequally resisting surface, by the rapidity of their development, by frequent attacks of acute pain from local inflammation of the cyst, and by the general symptoms and appearance of cancerous cachexy." 241.

Dr. Hope proceeds to the diseases of the uterus and its appendages. He successively adverts to uterine peritonitis—to inflammation of the uterine appendages, and of its proper muscular and mucous tissues—to inflammation and suppuration in the uterine veins and absorbents. We have recently adverted to these topics at such length, that we are compelled to quit our able author on his entrance into this interesting region.

The plates of these two Fasciculi are fully equal, if not superior, to any of their predecessors—a merit and an eulogy of no mean character. They consist of thirty-one figures, some of larger, some of contracted dimensions. They illustrate the subjects considered in the text. Cancer—hypertrophy of the submucous tissue—chronic peritonitis—the physiology of the Graafian vesicles and ova, and diseases of the ovaria—uterine peritonitis and phlebitis—and crural phlebitis are delineated in the plates before us.

It is difficult and almost needless to select particular instances for commendation. We will slightly allude to two or three drawings of interest or beauty.

Fig. 177 is a good representation of a striking case of stricture of the sigmoid flexure of the colon. The stricture was composed of a gristly substance resulting from hypertrophy of the submucous cellular tissue. The narrowest part of the stricture only permitted the introduction of a bougie one-third of an inch in diameter. The mucous membrane was thickened, tumid, lumpy, and vascular, filling up the passage so as to prevent the descent of water through it by its own gravity. The adipose tissue beneath the peritoneum was vascular and lax. The mucous membrane above and below the stricture was of brown colour from chronic inflammation.

The brief notes of the case are added by our author. They display the main features of what attracted much interest at the time of its occurrence.

"Mrs. Webb, æt. about 40, at St. George's Hospital, June, 1829, under Dr. Seymour; she was emaciated, and had had no alvine evacuation for a month. Intestinal tympanitis: severe bearing-down pains in the hypogastrium for two months; obstinate constipation for two years. A considerable tumour was felt projecting into the vagina between the cervix uteri and the rectum, and the same could be felt in the rectum. It suddenly disappeared when pressure was exercised upon it simultaneously per vaginam et rectum.

Ol. croton, &c., had no effect, and enemata could not be forced above the obstacle, even with the aid of a stomach-pump tube. Increase of pain and tympanitis; pulse very quick; anxiety; incoherence; death on the fifth day.

*Sectio.*—The tumour felt per vaginam et rectum was the right ovary, as large as a kidney, which had probably been forced into that situation by the inflated intestines, and was driven back again by the pressure with the fingers. The intestinal obstruction was occasioned by the stricture delineated in the Fig. seated about ten inches up the gut." lxx.

The 180th Figure is a beautiful example of areolar gelatiniform cancer of the stomach. For this Dr. Hope is indebted to Cruveilhier.

Dr. Hope incidentally remarks, that to areolar gelatiniform cancer, confluent in the adipose tissue, Mr. Abernethy gave the name of pancreatic sarcoma.

Fig. 185 is a fine drawing of ulcerated cancer of the mamma. It shews the co-existence of scirrhus and encephaloid disease, and many, if not most, of the stages of both. The patient died in a week after amputation of the breast, which was performed by Mr. Keate.

The history of a tumor displaying the characters ascribed by Mr. Abernethy to "vascular sarcoma" is not devoid of instruction.

"*Case.*—A woman, æt. 60, in the Edinburgh Infirmary. The tumour was seated in the middle of a puckered cicatrix, from which my friend, the late Mr. George Bell, informed me that he had removed a large ulcerating cancer fourteen years before. The present tumour, which had grown rapidly, was removed by Sir George Ballingall. Two years subsequently, when travelling in the Highlands, I accidentally met my old patient, and was sorry to find that a new tumour had recently begun to form in the same spot." lxxv.

The drawing of cirrhosis is well executed.

We can afford but a few lines more to Dr. Hope. We would direct particular attention to the delineations of the uterus and ovary of the recently impregnated female—to that of encephaloid tumour of the ovarium—to that of encysted tumours of the same organ—to that of uterine phlebitis—to those of crural phlebitis. The fear of appearing too encomiastic prevents our saying more.

The very brief space that remains compels us to defer a particular notice of Cruveilhier till our next. We shall then endeavour to lay before our readers a complete, and, we hope, an instructive account of this and other works that may appear, in the interim, on the subject of morbid anatomy.

---

We have little space left for even commendation of the labours of our military brethren at Chatham. Some statements, cheering to the lover of the profession, flattering to the parties immediately concerned, are contained in the preface to the drawings.

"The collection having received great and many additions, a descriptive Catalogue of it was published a few months ago for the use of the Medical Officers; and in now publishing a Second Fasciculus, it will be observed that reference is made in the Plates to short descriptions of the preparations enumerated in that Catalogue: but besides these short descriptions, a much more extensive Manuscript Catalogue exists at Chatham; and further, there are deposited in the Library there, fully detailed cases of patients from whom preparations have been made, and comprised in not fewer than 300 folio volumes of Clinical histories, and to which, as well as to the preparations, ready reference may at all times be had."

"There are in the Anatomical Museum at Chatham abundant materials for continuing the Work when it can be done with advantage. They are

1420 Preparations in Morbid Anatomy.
500 Preparations in Natural Anatomy.
372 Preparations in Comparative Anatomy.
500 Paintings, Drawings, Casts, &c.

The Library of the Medical Department, commenced in 1822, now contains nearly 2600 volumes."—*Preface.*

The first plate is illustrative of phthisis laryngea—the false membrane formed in croup—the sequelæ of varioloid pustules in the larynx—and the consecutive stages of the pseudo-membranes consequent on inflammation of the pleuræ. The third plate elucidates several diseases of the lungs. It is managed well. The fourth plate exhibits a beautiful view of the effects of pericarditis. The adhesions of the pericardium are shown, and should be remembered, as a consequence of this affection. A hypertrophic heart and an aneurism of the abdominal aorta form the main subjects of the fifth plate. Scirrhus and dissolution of the stomach occupy the sixth. The seventh is devoted to the pathology of the mucous and the peritoneal membrane of the intestines. The morbid anatomy of dysentery, and scirrhus of the ileo-colic valve are figured in the eighth. The ninth and last displays an enormous abscess in the liver—hydatids—melanosis, and what is denominated the *tuberculated liver.* With an abstract of a short memorandum on the latter we lay aside the pen. Yet we cannot do so without an earnest recommendation to our affluent professional brethren, to scientific bodies, and to book societies, to encourage the works of which we now take leave.

"The term tuberculated (says the compiler of the descriptions of the plates) might be changed with propriety as the bodies delineated (of a firm texture and brownish or dirty yellow colour,) have not the characters of the objects generally denominated tubercles: it is, however, retained here for want of a better. What the exact composition or nature of these tubercles is, does not seem to have been yet satisfactorily explained. Some suppose them to be produced by hypertrophy of the white substance of the Liver, others by a new deposit; but from the examination of a series of specimens in this Museum, showing their progressive stages of development, it appears as likely that they consist of an alteration of the red substance of the organ combined with, probably, some new deposition. Livers containing these bodies are usually very small and hard, and jaundice with ascites are generally attendant symptoms."

